# Vascularized cardiac tissue construction with orientation by layer-by-layer method and 3D printer

**DOI:** 10.1038/s41598-020-59371-y

**Published:** 2020-03-26

**Authors:** Yoshinari Tsukamoto, Takami Akagi, Mitsuru Akashi

**Affiliations:** Building Block Science Joint Research Chair, Graduate School of Frontier Biosciences, Osaka University, 1-3 Yamadaoka, Suita, 565-0871 Japan

**Keywords:** Cardiovascular models, Extracellular matrix

## Abstract

Herein, we report the fabrication of native organ-like three-dimensional (3D) cardiac tissue with an oriented structure and vascular network using a layer-by-layer (LbL), cell accumulation and 3D printing technique for regenerative medicine and pharmaceutical applications. We firstly evaluated the 3D shaping ability of hydroxybutyl chitosan (HBC), a thermoresponsive polymer, by using a robotic dispensing 3D printer. Next, we tried to fabricate orientation-controlled 3D cardiac tissue using human induced pluripotent stem cell-derived cardiomyocytes (hiPSC-CM) and normal human cardiac fibroblasts (NHCF) coated with extracellular matrix (ECM) nanofilms by layer-by-layer technique. These cells were seeded in the fabricated rectangular shape HBC gel frame. After cultivation of the fabricated tissue, fluorescence staining of the cytoskeleton revealed that hiPSC-CM and NHCF were aligned in one direction. Moreover, we were able to measure its contractile behavior using a video image analysis system. These results indicate that orientation-controlled cardiac tissue has more remarkable contractile function than uncontrolled cardiac tissue. Finally, co-culture with human cardiac microvascular endothelial cells (HMVEC) successfully provided a vascular network in orientation-controlled 3D cardiac tissue. The constructed 3D cardiac tissue with an oriented structure and vascular network would be a useful tool for regenerative medicine and pharmaceutical applications.

## Introduction

Tissue engineering technology uses living cells, cytokines and biomaterials with the primary goal of creating functional tissue substitutes for novel medical treatments and pharmaceutical development^1^. In the human body, native organs and tissues such as heart, lung, liver and kidney have very complex three-dimensional (3D) structures with highly specialized cellular and extracellular matrix (ECM) components. For example, cardiac tissue has an oriented structure in which cells are aligned in one direction. In the heart, cardiomyocytes have different orientations for each layer, allowing it to produce a large contractile force and stimulus transmission function to deliver blood to the whole body^2,3^. Therefore, fabrication of 3D tissue with a structure and function similar to native organs for application to medical treatment and pharmaceutical development is required. In order to achieve this, biofabrication technology such as bioprinting and bioassembly for controlling cells and materials is important^4^. Among them, cardiac tissue is one of the important organs for life support^5^. For this reason, human cardiomyocytes (CM) are difficult to use for fabrication of 3D-CM tissues *in vitro*. Due to recent advances in the development of human induced pluripotent stem cells (hiPSC) and methods to differentiate them to CMs, they have become available as a cell source for the fabrication of 3D cardiac tissue^6,7^. For the development of novel cell transplantation therapy and drug screening *in vivo*, it is important to fabricate cardiac tissue with the heart-specific structure of orientation in order to complement organ function.

Many researchers have reported the development of 3D cardiac tissue by using scaffolds such as synthetic substrates and natural macromolecules^8–12^. In addition, other researchers have developed scaffold-free technologies^13–15^, cell sheet engineering^16–20^, and decellularized techniques^21–25^. These methods are high-potency systems of 3D cardiac tissue fabrication. In particular, there have been some reports on the fabrication of orientation-controlled cardiac tissue^12,26–30^. Morimoto *et al*. have reported a fabrication method of orientation-controlled cardiac tissue by patterning of the hydrogel structures with hiPSC-CM^30^. Moreover, they demonstrated the drug reactivity of fabricated tissue, the changes beating rate and contractile force. Li J *et al*. reported the fabrication of cardiac tissue-like constructs by cultivating hiPSC-CM on aligned nanofibers. They also reported the repair of a myocardial infarction model rat’s heart when these tissues were engrafted^8^. On the other hand, cardiac tissues require blood vessels because they are thick and have high cell density thus consuming a large amount of nutrients and oxygen. However, reports about fabrication of 3D cardiac tissue with an oriented and vascularized structure are seldom mentioned and have many problems such as the lack of bottom-up technology. In order to solve this problem, the 3D printer technology which precisely controls the placement of cells and materials is attracting great attention^31–36^.

**Figure 1:**
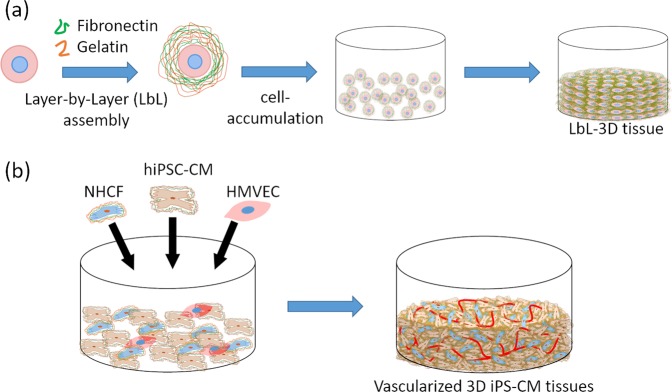
(**a**) Schematic illustration of the fabrication process for layer-by-layer (LbL) 3D tissue using fibronectin (FN) and gelatin (G) coating technique and cell accumulation technique. (**b**) Schematic illustration of fabrication of 3D cardiac tissue with a blood capillary network using LbL coated cells and cell accumulation technique.

In our previous study, we developed a fabrication approach of 3D multilayered tissue, “a hierarchical cell manipulation technique”, by coating nanometer-sized ECM films of fibronectin (FN) and gelatin (G) onto a cell surface using layer-by-layer (LbL) assembly^37^. In addition, we also developed a rapid bottom-up technique, “cell accumulation technique”, by a single cell coating using FN-G nanofilms (Fig. 1a)^38^. We have demonstrated the fabrication of various kinds of 3D tissue models such as skin models^39,40^, blood vessel models^41^, blood/lymph-vascularized cancer metastasis models^42^, pancreas models^43^, liver models^44,45^ and heart models^46^. Primarily, the heart model was fabricated by using hiPSC-CM and has a vascular network (Fig. 1b). This model was used to evaluate drug response with respect to cardiotoxicity *in vitro*. Moreover, this vascularized cardiac model was applied to an animal transplantation experiment^47^. However, these models have yet to possess orientation-controlled tissue which is specific to the structure of the heart. It is important to control the orientation of cells for the successful fabrication of functional 3D cardiac tissue models.

**Figure 2:**
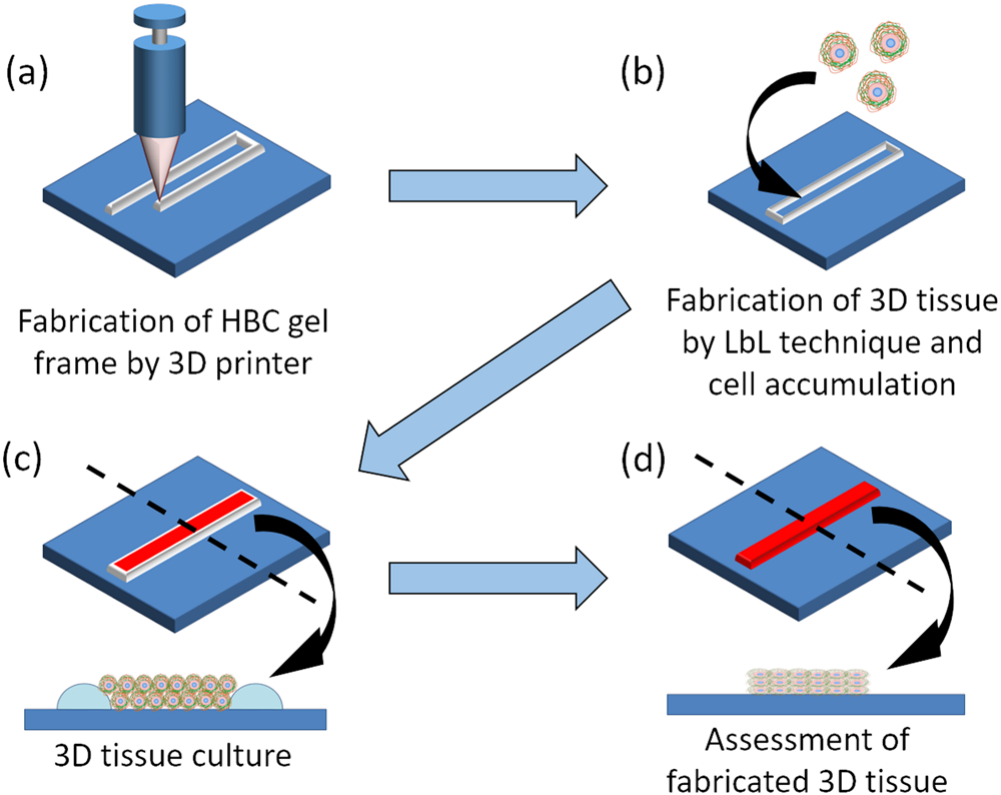
Schematic illustration of fabrication of orientation-controlled 3D cardiac tissue using 3D printing technology. (**a**) 3D printing of HBC using a robotic dispensing 3D printer. (**b**) Fabrication of 3D multilayer tissue using LbL coated cells and cell accumulation technique. (**c**) Cultivation of orientation-controlled 3D tissue. (**d**) Assessment of shape and contractile properties using a histological technique and image processing.

Here, we report on a method for the fabrication of 3D cardiac tissue with heart specific structure, cell orientation and vascular network. To achieve this purpose, we reported on a fabrication method of orientation-controlled 3D tissue by using an LbL technique, cell accumulation method and 3D print technology^48^. The cell direction in the 3D tissue was aligned by controlling the 3D tissue shape using a 3D printed gel frame. By controlling the shape of the tissue linearly, the cells in the 3D tissue are directionally influenced by patterning and tensile force. In this report, we applied the orientation control method for fabrication of functional 3D cardiac tissue (Fig. 2). At first, we fabricated the gel frame on a culture insert for control cell orientation by using a 3D printer and a thermo-responsive polymer gel, hydroxybutyl chitosan (HBC). HBC has the ability of sol-gel transition depending on the temperature. In the next step, we fabricated ECM nanofilms onto hiPSC-CM and normal human cardiac fibroblast (NHCF) cells by using the LbL technique. In addition, these cells were seeded in the HBC gel frame by using the cell accumulation method to make orientation-controlled 3D tissue. After culture, cell morphologies and contraction function of the fabricated 3D tissues were evaluated by using fluorescent staining and image analysis^49–51^. Finally, we tried to fabricate native-like 3D cardiac tissue with orientation and vascular network constructs using co-cultured hiPSC-CM, NHCF and human cardiac microvascular endothelial cells (HMVEC).

## Results

### Assessment of HBC gel printing by robotic dispensing 3D printer

**Figure 3:**
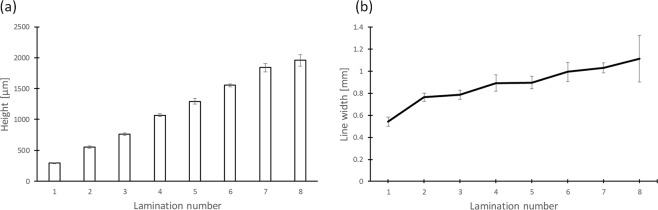
Observation and analysis of a laminated 5% HBC gel wall printed by a robotic dispensing 3D printer. (**a**) Height of laminated HBC gel wall observed from the horizontal direction. (**b**) Line width of laminated HBC gel wall observed from the vertical direction.

We tried to assess the shaping ability of the robotic dispensing 3D printer for printing an HBC gel 3D structure using HBC as an ink material. The ink cooled to 4 °C by a Peltier element in the ink tank was printed and assembled into a linear shape on a glass plate heated to 50 °C. The results of this assessment were that the HBC line width was about 1 mm and the HBC gel could be laminated up to 8 layers. Figure 3a shows the measured height of the assembled HBC gel wall. The HBC gel wall height increased from 300 μm to 2 mm as the lamination number increased from 1 to 8. Figure 3b shows the width of the HBC gel wall. The HBC gel wall width increased from 500 μm to 1.1 mm.

### Fabrication of orientation-controlled 3D cardiac tissue by HBC polymer gel

**Figure 4:**
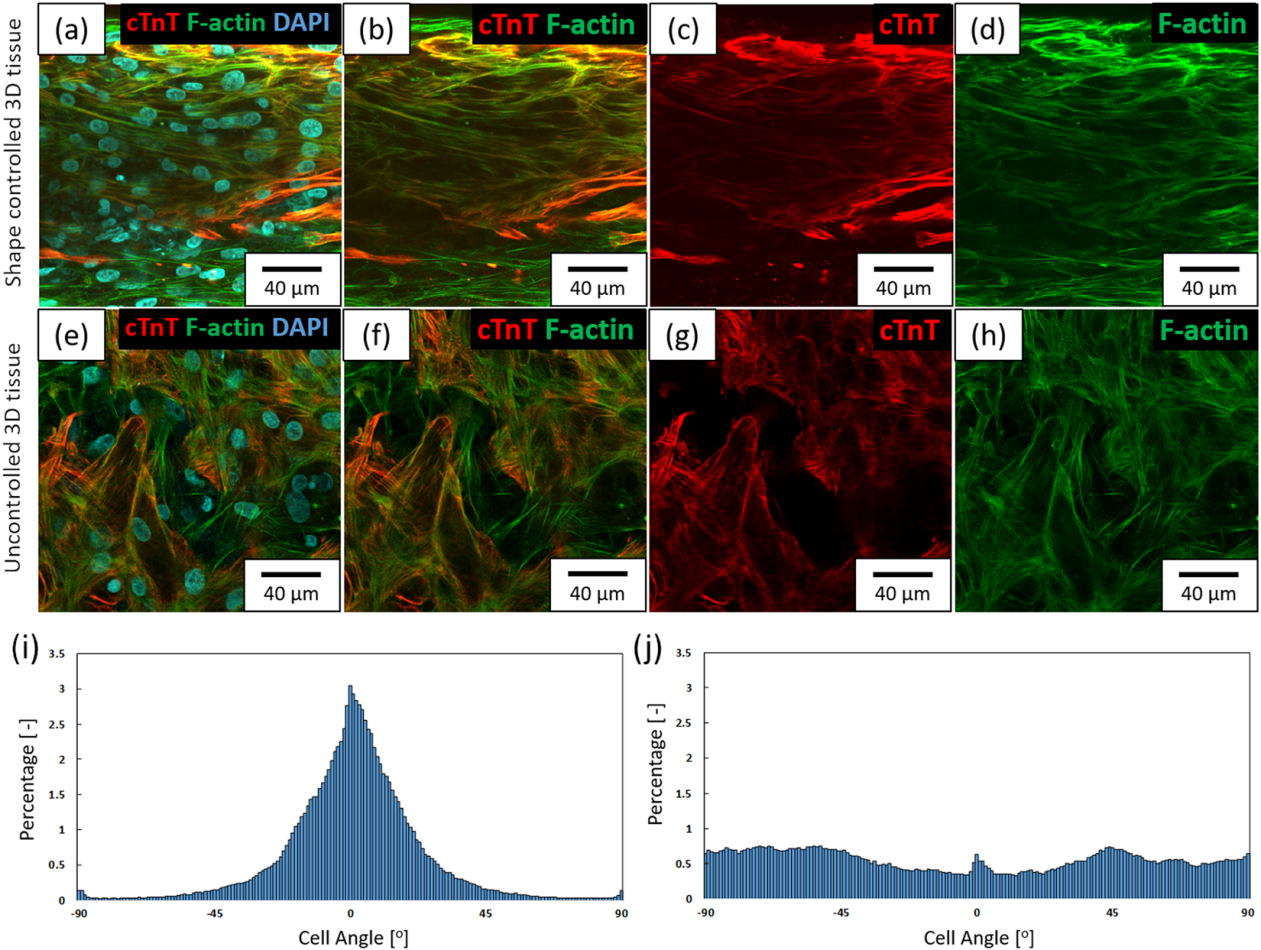
Shape controlled 3D cardiac tissue image stained with fluorescent labeling phalloidin and anti-cardiac troponin T (cTnT) antibody obtained from confocal laser scanning microscopy (CLSM). (**a–d**) Shape controlled 3D cardiac tissue using a 2 × 15 mm HBC gel frame. (**e–h**) Uncontrolled 3D cardiac tissue. (**a,e**) The merged images of F-actin, cTnT and DAPI. (**b**,**f**) The merged images of F-actin and cTnT. (**c,g**) The cTnT images. (**d,h**) The F-actin images. (**i,j**) The graphs of the local alignment angles of F-actin fibers in shape controlled 3D cardiac tissue are shown underneath the CLSM image by image analysis.

We tried to fabricate orientation-controlled 3D cardiac tissue by printed HBC gel frame. In a previous study, we fabricated orientation-controlled 3D tissue of NHCF using a shape controlled rectangular shaped HBC gel frame with a short side of 2 mm or less^48^. We deduced that orientation can be controlled by limiting the direction of extension and direction of tension by forming the tissue thin. In this study, we focused on the rectangular-shaped HBC gel for fabrication of the orientation-controlled 3D cardiac tissue. The HBC gel was printed onto a culture insert by using the robotic dispensing printer^48^. The fabricated HBC gel frame shape was rectangular with a long side of 15 mm and a short side of 1.5 mm. Figure 4a–d shows a fabricated shape-controlled 3D cardiac tissue by printed HBC gel frame with a 1.5 × 15 mm rectangle (The overview image was shown in Supplementary Fig. 1). Figure 4e–h shows an uncontrolled 3D cardiac tissue by culture in a 24-well insert. Figures 4a–h show the fluorescent staining image of fabricated 3D cardiac tissue. Figure 4c,g show cTnT, a known cardiac marker, in fabricated 3D cardiac tissue stained with Alexa 546 conjugated antibody. Figure 4d,h show F-actin of NHCF and cardiomyocytes in fabricated 3D cardiac tissue stained with Acti-stain 488 fluorescent phalloidin (See Supplementary Fig. 2). Figure 4i,j show the assessment result of the cell orientation obtained from F-actin images by ImageJ software. Cell orientation was calculated from the F-actin fiber angle of NHCF and cardiomyocytes. These graphs show 3D cardiac tissue by HBC gel frame with a 1.5 × 15 mm rectangle (Fig. 4i) and 24-well culture insert (Fig. 4j). From these data, the number of cells oriented in the same direction in the 3D cardiac tissue by HBC gel frame with a 1.5 × 15 mm rectangle was higher than the 3D cardiac tissue in 24-well culture insert.

### Contractile properties of fabricated 3D cardiac tissues

**Figure 5:**
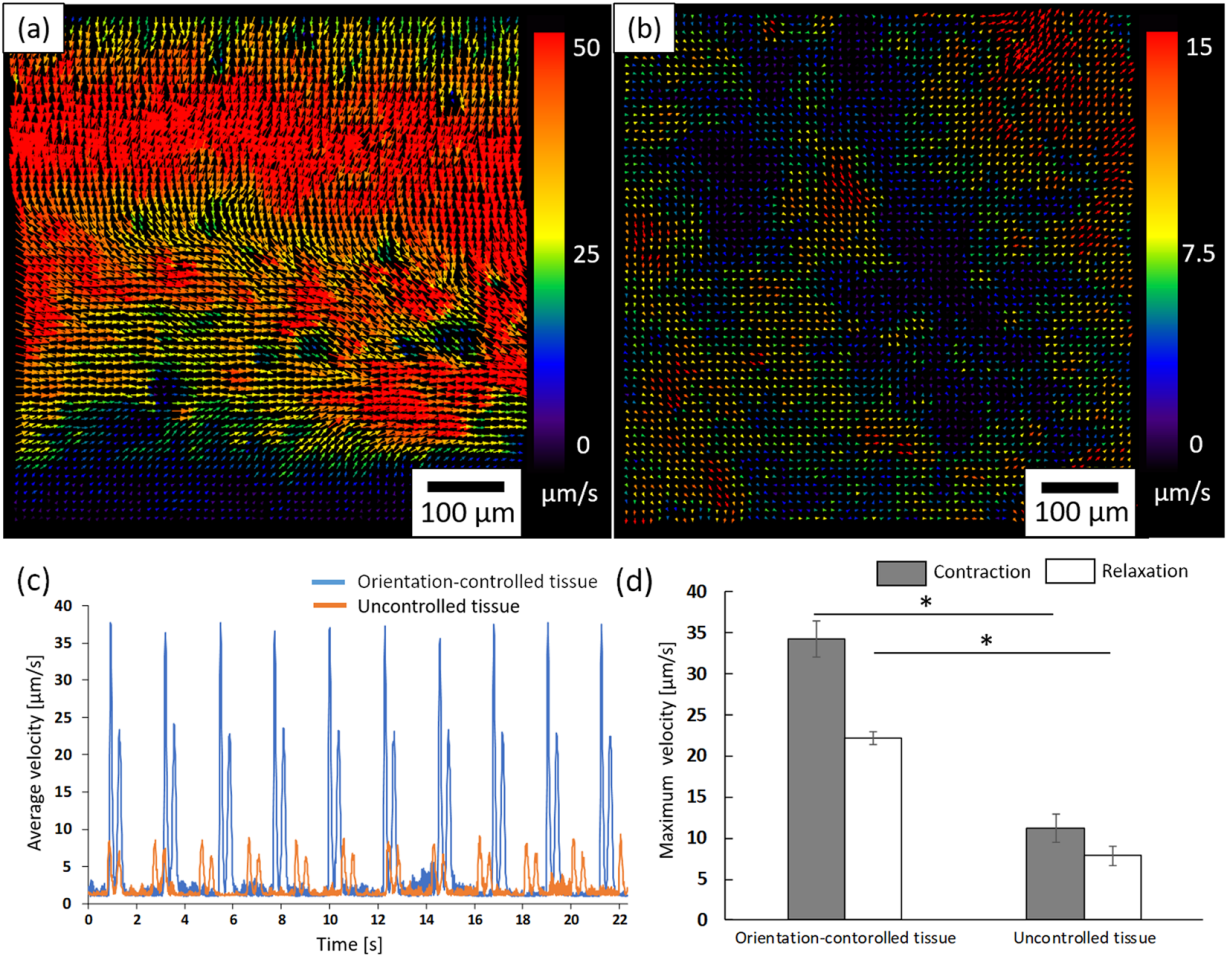
The motion analyses of surface movement of 3D cardiac tissue calculated by particle image velocimetry (PIV). (**a**) The motion analysis result of orientation-controlled 3D cardiac tissue. (**b**) The motion analysis result of orientation uncontrolled tissue. The arrows indicate the direction of movement of the separated 21 μm square block and the colors show the velocity of the movement. The color scales are as indicated beside each of the figures. (**c**) Plot of a motion waveform representing contraction and relaxation peaks. The blue plot indicates the orientation-controlled tissue. The orange plot indicates the uncontrolled tissue. (**d**) The graph of maximum contraction and relaxation velocity. (*p < 0.05, n = 3).

Next, we tried to assess the contractile properties of fabricated 3D cardiac tissues using an image analysis technique. Figure 5a,b show the result of the motion vector images calculated from phase contrast images of orientation-controlled 3D cardiac tissue (Fig. 5a) and uncontrolled 3D cardiac tissue (Fig. 5b) (The original movie is shown as Supplementary Movies 1 and 2). The arrows’ direction and color indicate the contraction direction and the contraction speed of the 3D cardiac tissues. From these results, the cardiomyocytes of orientation-controlled 3D tissue moved in the same direction and synchronized a large number of cells. In the case of uncontrolled 3D cardiac tissue, cardiomyocytes moved in different directions and the contracting area was smaller than in the orientation-controlled 3D cardiac tissue. Figure 5c shows the average velocity and time intervals of orientation-controlled 3D cardiac tissues (blue) and uncontrolled 3D cardiac tissues (orange). From these data, it was established that the contraction velocity of orientation-controlled 3D cardiac tissue was faster than uncontrolled tissues. Figure 5d shows the difference between orientation-controlled tissue and uncontrolled tissue in maximum contraction speed and relaxation speed. From these data it was established that orientation-controlled tissue contracted and relaxed twice as quickly as uncontrolled tissue.

### Fabrication of the orientation-controlled 3D cardiac tissue with vascular network

**Figure 6:**
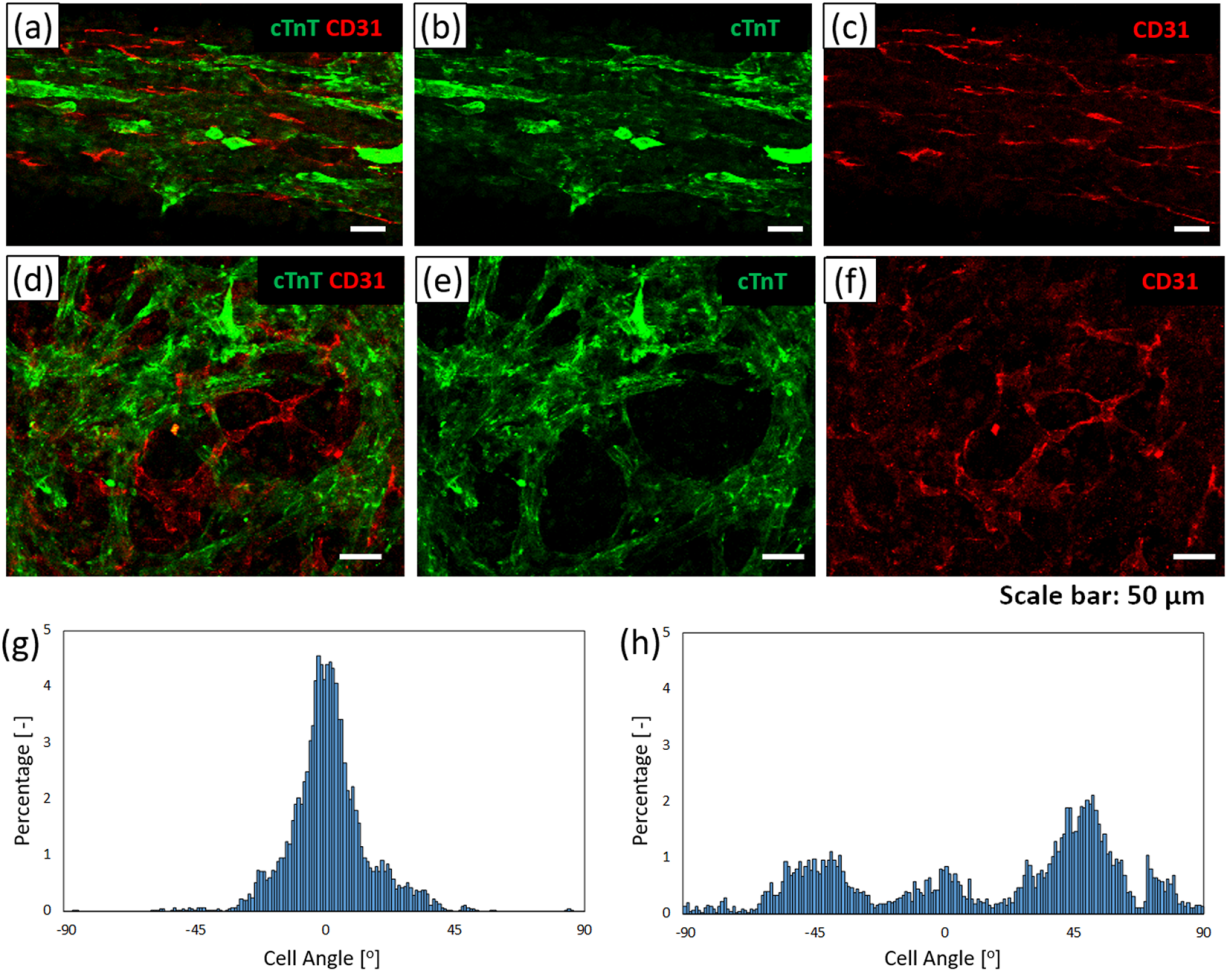
Shape controlled 3D cardiac tissue with vascular network image stained anti-cardiac troponin T (cTnT) antibody and anti-CD31 antibody obtained from LSCM. (**a–c**) Shape controlled 3D cardiac tissue using a 1.5 × 15 mm HBC gel frame. (**d**,**e**,**f**) Uncontrolled 3D cardiac tissue. (**a,d**) The merged image of cTnT (green) and CD31 (red). (**b,e**) The cTnT image. (**c,f**) The CD31 image. (**g,h**) The graphs of the local alignment angles of vascular network (CD31) in shape controlled 3D cardiac tissue are shown underneath the CLSM image by image analysis. (**g**) The graph of orientation-controlled tissue. (**h**) The graph of uncontrolled tissue.

Finally, we constructed orientation-controlled 3D cardiac tissues with vascular network. To introduce the vascular network into the 3D cardiac tissue, hiPSC-CMs and NHCF coated FN-G nanofilms by LbL technique were co-cultured with HMVEC in a 1.5 × 15 mm rectangular HBC gel frame (5%). After 5 days of culture, hiPSC-CM and HMVEC in the fabricated 3D cardiac tissues were fixed and stained by immunofluorescence stain. Figure 6b,e show cTnT images of hiPSC-CM stained with anti-cTnT antibody to investigate the distribution of hiPSC-CM in 3D cardiac tissue. Figure 6c,f show CD31 images of HMVEC stained with anti-CD3l, a specific endothelial marker stained to investigate the shape and distribution of the vascular network. Figure 6a–c show a fabricated shape-controlled 3D cardiac tissue by printed HBC gel frame with a 1.5 × 15 mm rectangle. Figure 6d–f show an uncontrolled 3D cardiac tissue by culture in a 24-well insert. As a result of hiPSC-CM, NHCF and HMVEC co-culture, the orientation of hiPSC-CM could be controlled by using a 1.5 mm short side rectangular HBC gel frame. Figure 6g,h show the assessment result of the vascular network orientation obtained from CD31 images by ImageJ software. Cell orientation was calculated from vascular network angle of HMVEC. From these data, vascular network has orientation structure in the orientation-controlled 3D cardiac tissue, but not has orientation structure in the uncontrolled 3D cardiac tissue.

## Discussion

Artificial 3D tissues are in great demand in regenerative medicine and the drug development field for medical treatments and *in vitro* assays. In the fabrication of artificial 3D tissues, it is necessary to regulate the microenvironment such as placement of multiple cells and ECM. This is because the organs and tissues in the human body appeared to have multiple functions and specific structures such as 3D structure, cell-ECM interaction and cell-cell interaction. We have reported a fabrication method of 3D tissue with only cells and ECM using an LbL technique and cell accumulation technique. This technique is able to control the cell type per layer and construct blood capillaries in the 3D tissue. It has the advantages of high cell density for cell-cell interaction and early vascular network formation compared with other methods of 3D tissue fabrication, such as using porous scaffolds, nanofiber scaffolds and hydrogels. The fabrication of biomimetic 3D tissue with a multilayered structure like skin was easily achieved using an LbL and cell accumulation technique. However, the reproduction of complex structures such as orientation of muscle cells and polarity of hepatocytes was difficult by this technique. In our previous study, we could fabricate the 3D cardiac tissue by hiPSC-CM and use it for drug evaluation. In addition, we reported a fabrication method of orientation-controlled 3D tissue by 3D printing technology^48^. The cells in a linearly controlled 3D tissue are aligned in one direction by the influence of patterning and tensile force. As known, cells recognize the surrounding environment such as topography and tensile force from other cells. In the case of 3D tissue fabrication, cells are able to have an oriented structure by recognizing topography and tensile force like 2D culture. In this study, we tried to fabricate orientation-controlled 3D cardiac tissue by using an LbL technique, cell accumulation technique and 3D printing technology.

At first, we assessed the capability of 3D modeling by using a robotic dispensing 3D printer and HBC gel. The HBC gel could be assembled to at least 8 layers with a linear shape (Fig. 3a). A ninth layer could not be laminated because the HBC gel wall melted. The reason for this is that the ninth layer is far from the substrate and melts because it cannot receive temperature control. From our previous studies, however, the thickness of 3D tissue is limited to 100 μm. For this reason, the 3D modeling ability of HBC gel is sufficient to fabricate 3D tissue using an LbL technique and cell accumulation technique. In addition, HBC gel has the ability of sol-gel transition in response to temperature. For this reason, only the HBC gel can be removed by cooling and only the fabricated 3D tissue can be collect.

Next, we fabricated orientation-controlled 3D cardiac tissue by using 3D printing of HBC gel, LbL technique and cell accumulation technique. In our previous study, we were able to fabricate orientation-controlled 3D fibroblast tissue using these techniques^48^. When the 3D tissue shape was controlled by the printed HBC gel frame with a 2 × 5 mm rectangle shape, the cells in the 3D tissue extended in one direction. Based on this result, we tried to fabricate orientation-controlled 3D cardiac tissue. We observed the morphologies of hiPSC-CM and NHCF stained with F- actin and cTnT by fluorescence dye in fabricated 3D cardiac tissue (Fig. 4a–h). The assessment result of the cell orientation obtained from F-actin images (Fig. 4i,j) indicated that the cell orientation could e controlled in the case of cardiomyocytes by using a printed HBC gel frame with a 1.5 × 15 mm rectangle. Comparing the fabricated 3D cardiac tissue with the native heart tissue, the cell density in native heart tissue is 10^8–9^ cells/cm^3 ^^52,53^. On the other hand, the cell density of the 3D cardiac tissue fabricated by the tissue fabrication method by the LbL technique used in this study was about 6 × 10^8^ cells/cm^3^ calculated. Native heart tissue has the orientation structure and different orientation in each layer^54^. On the other hand, although the 3D cardiac tissue fabricated by LbL technique can not be achieved yet, it has a unidirectionally oriented structure. This result is similar to our previous study and indicates that these techniques are suitable methods for fabrication of orientation-controlled 3D cardiac tissues.

Moreover, we tried to assess the contractile properties of fabricated 3D cardiac tissues by image analysis. Figure 5a,b indicate that orientation-controlled 3D cardiac tissue contracted in one direction compared to uncontrolled tissue. In addition, Fig. 5c shows that the contraction speed of orientation-controlled 3D cardiac tissue was faster than the uncontrolled one. Figure 5d indicates the quantitative value of maximum contraction and relaxation speed obtained from a plot of the average velocity and time intervals. From this quantitative value, orientation-controlled tissue contracts more than twice as quickly as uncontrolled tissue. In the heart of the living body, the orientation of cardiomyocytes is known to be an important factor for producing a large contractile force and stimulus transmission function^2,3^. For these reasons, this assessment result indicates that it is possible to fabricate cardiac tissue with a function similar to a living body using the techniques outlined in this study.

Finally, we tried to fabricate orientation-controlled 3D cardiac tissue with a vascular network by using an HBC gel frame with a 1.5 × 15 mm rectangle. Vascular network is important structures in the fabrication of 3D tissue for use in regenerative medicine and drug development fields which require thicker tissue. The reason for the necessity of blood vessels is that oxygen diffusion is limited to a depth of about 100 μm from the tissue surface^55^. If there are no blood vessels, the inner cells of 3D tissues that are thicker than 100 μm become necrotic due to lack of oxygen^56^. For this reason, we developed a fabrication method for 3D cardiac tissue with vascular network. Figure 6a–f show the hiPSC-CM stained cTnT and HMVEC stained CD31 immunofluorescence staining images obtained from orientation-controlled cardiac tissue (Fig. 6a–c) and uncontrolled cardiac tissue (Fig. 6d–f). From the result of CD31 stained images, vascular network formed in both tissues. In the case of orientation-controlled tissue, the vascular network has an oriented structure similar to cardiomyocytes according to image analysis (Fig. 6g). In the case of uncontrolled tissue, on the other hand, the vascular network does not have an oriented structure (Fig. 6h). Rosenfield *et al*. reported on the orientation of vascular network in a 3D gel structure^57^. They indicated that the orientation of vascular network was formed by tensile force in the 3D tissue. In our study, we consider that vascular network orientation in the fabricated 3D cardiac tissue was caused by tensile force which regulated the orientation in one direction. However, in order to fabricate thicker and functional tissue, perfusion culture utilizing vascular network is required. Therefore, we work on tissue fabrication using perfusion culture. The obtained data suggest that orientation-controlled 3D cardiac tissue with vascular network would be efficacious in transplantation therapy and drug assessment.

We revealed that orientation-controlled tissue made by this study has native organ like structure and high function. We are conducting research to apply this tissue as a more effective therapeutic effect than conventional tissue. Moreover, since it is expected to exhibit reaction similar to native organ, we are working on application as a drug discovery model. Additionally, it is expected to be applied to tissues having oriented structures such as cardiac tissue as well as skeletal muscle, nerve and vascular tissue. From the above, this research is considered to contribute greatly to regenerative medicine and drug discovery research.

In conclusion, we successfully fabricated 3D cardiac tissues with an oriented structure and vascular network using an LbL technique, cell accumulation technique and 3D printing technology using a thermoresponsive polymer gel as an ink material. The fabricated orientation-controlled 3D cardiac tissue had an oriented structure and exhibited better contractile properties than the uncontrolled tissue. The controlled tissue contracted in one direction and showed a high speed of contraction. Moreover, the orientation-controlled 3D cardiac tissue has a vascular network. This 3D cardiac tissue has the potential for usage in transplantation medical care and drug assessment because it has the native heart organ-like structure and vascular network for the fabrication of thicker and larger 3D tissue. Therefore, we believe that the 3D cardiac tissue with orientation and vascular network would be a useful tool for regenerative medicine and pharmaceutical applications.

## Materials and Methods

### Materials

All of the chemical reagents were used without further purification. Chitosan was kindly donated by Dainichiseika Color & Chemicals Mfg. Co. Ltd (Tokyo, Japan). Fibronectin from human plasma, Triton X-100, human recombinant FGF-2, 1-thioglycerol and bovine serum albumin were purchased from Sigma-Aldrich (MO, USA). Gelatin, L-ascorbic acid 2-phosphate trisodium salt, IWR-1-endo and Y-27632 were purchased from Wako Pure Chemical Industries (Osaka, Japan). fetal bovine serum, 4′6-diamidino-2-phenylindole, dihydrochloride (DAPI), Alexa Fluor 546-conjugated goat anti-rabbit or anti-mouse IgG cross-absorbed secondary antibody, Alexa Fluor 488-conjugated goat anti-mouse IgG cross-absorbed secondary antibody, Knockout-DMEM/F12, knockout serum replacement, 2-mercaptoethanol, MEM non-essential amino acids, L-glutamine and StemPro-34 were purchased from Thermo Fisher Scientific (MA, USA). Cell culture inserts with a 0.4 μm pore membrane (polyester) and cell culture inserts with a 3 μm pore membrane (polycarbonate) were purchased from Corning (NY, USA). NHCF, HMVEC, fibroblast growth medium (FGM-3), and endothelial growth medium (EGM-2 MV) were purchased from Lonza (Basel, Switzerland). Acti-stain 488 fluorescent phalloidin and Acti-stain 555 fluorescent phalloidin were purchased from Cytoskeleton, Inc (CO, USA). Rabbit polyclonal anti-CD31 antibody and anti-cardiac troponin T (cTnT) antibody were purchased from Abcam (Cambridge, UK). The monoclonal mouse anti-human CD31 antibody was purchased from Dako (Glostrup, Denmark) (CA, USA). Dulbecco’s modified eagle medium (DMEM), Antibiotic-antimycotic mixed stock solution and 4% paraformaldehyde (PFA)/PBS were purchased from Nacalai Tesque (Kyoto, Japan). 1,2-butylene oxide (BO) was purchased from Tokyo Chemical Industry Co., Ltd (Tokyo, Japan). Human iPS cell line 253G1 was obtained from RIKEN Bio Resource Center (Ibaraki, Japan). Mitomycin C-inactivated mouse embryo fibroblast feeder cells, dispase II and VEGF165 were purchased from Millipore Co. (MA, USA), Roche Diagnostics (Basel, Switzerland) and HumanZyme (IL, USA), respectively. Recombinant human proteins of BMP-4 and activin A were purchased from R&D systems Inc (MN, USA). Accutase and Accumax ware purchased from Innovative Cell Technologies, Inc (CA, USA). IWP-2 was purchased from TOCRIS Bioscience (Bristol, UK).

### Synthesis of thermoresponsive polymer

We used HBC gel that has a thermoresponsive function as an ink material for the 3D printer. HBC gel is known to have the ability of sol-gel transition in response to temperature. In this study, we synthesized HBC gel using a previously reported method^58^. Chitosan polymer was dissolved into 0.1 M hydrogen chloride (HCl). Sodium hydroxide (NaOH) (5 M) was added into the chitosan solution to adjust the pH to 8. After warming the chitosan solution to 85 °C, BO was added and it was mixed for 3 h. Some sediment of HBC appeared during the reaction. The sediment was dissolved with 5 M HCl. Next, another BO was added and the reaction was continued at 85 °C for 24 h. The product was then purified by dialysis in pure water at room temperature for 24 h and the purified product was collected by lyophilization for 4 days. The degree of substitution (DS) of the polymer was determined by elemental analysis referring to a previous report^59^.

The DS was computed by the following equation: DS = {(C/N)_m_-(C/N)_0_}/4, where (C/N)_m_ indicated C/N (mole ratios) of the chitosan derivative, (C/N)_0_ indicated the C/N (mole ratios) of the chitosan^59^.

### Cell preparation

Human iPS cells 253G1^60^ ware cultured on a Mitomycin C-treaded mouse embryonic fibroblast feeder layer in KnockOut Serum Replacement (KSR) based medium supplemented with 4 ng/ml FGF-2. KSR-based medium consisted of knockout-DMEM/F12 medium, supplemented with 20% (v/v) KSR, 0.1 mM 2-mercaptoethanol, MEM non-essential amino acids, and 2 mM L-glutamine. hiPSC were passaged every 7 days as small clumps by treatment with 1 mg/ml dispase II, followed by pipetting.

For hiPSC-CM differentiation, hiPSC clumps after accutase treatment, pipetting and 100 μm cell strainer (Corning, NY, USA) were suspended in 30 ml StemPro-34 medium containing 50 μg/ml L-ascorbic acid 2-phosphate trisodium salt, 2 mM L-glutamine and 400 μM 1-thioglycerol, and induce differentiation added 10 μM Y-27632, 10 ng/ml BMP-4, 5 ng/ml FGF-2, 3 ng/ml activin A. Cell suspension were seeded into a 30 ml single-use bioreactor (ABLE Corporation & Biott Co., Japan) at 37 °C, an agitation rate of 55 rpm. The culture medium was exchange containing: 10 ng/ml BMP-4, 5 ng/ml FGF-2 and 3 ng/ml activin A on day 2; 4 μM IWR1 and 10 μM IWP-2 on day 3 and day 5; 5 ng/ml VEGF and 10 ng/ml FGF- 2 on day 6, 8, 10 and 12^61^.

On day 13, hiPSC-CM were dissociated spheroids into single cell suspensions by Accumax treatment and disruption through repeated pipetting and 40 μm cell strainer (Corning, NY, USA). The single cells of hiPSC-CM were suspended in DMEM with 10% FBS. The cTnT positive rate of the used cardiomyocytes was more than 90%. In addition, in this study, cardiomyocytes were used without purification^61^.

NHCF (passage was less than 7) were cultured with FGM-3 and HMVEC (passage was less than 7) were cultured with EGM-2 MV at 37 °C in an incubator at 5% CO_2_.

### Designing 3D frames with a thermoresponsive polymer using a 3D printer

We used a dispenser type 3D printer (SHOTMASTER 200DS; Musashi Engineering) for printing the thermoresponsive polymer and fabricating the 3D structure (See Supplementary Fig. 3 and Supplementary Movie 3). Figure 2a shows a schematic of the robotic dispenser type 3D printer. This printer has a dispensing nozzle consisting of a 1 mL syringe with a double thread screw taper nozzle with an internal diameter of 610 μm. In addition, this printer has other modules such as a syringe pump module, Y moving stage and the X-Z moving motors module and temperature control module with a Peltier element. By using the temperature control module, the ink tank was cooled to 4 °C and the stage was heated up to 50 °C. The HBC (50 mg/mL) solution was loaded into the ink tank. In this study, the syringe pump, moving speed, and nozzle-to-collector distance were set at 7 μL/s, 2.5 mm/s, and 250 μm/layer respectively. After the HBC gels print, we assessed the shape of the printed HBC gels by observing them from a horizontal angle and measuring the laminated HBC gel height by ImageJ software^62^. We prepared the 6-well culture insert with a 0.4 μm pore membrane and the HBC gel was printed onto the membrane (nozzle cooled to 4 °C, stage heated up to 50 °C). Finally, we fabricated the HBC gel with a rectangular shape where the long side was 15 mm and the short side was 2 mm and 3 mm frame onto 6 well culture insert.

### Fabrication of ECM nanofilm using a filtration-LbL method

Isolated hiPSC-CM and NHCF were coated with FN-G nanofilms by filtration LbL method according to our previous report^46^. For the filtration-LbL, we prepared 2.5 mL of 0.2 mg/mL FN in PBS and G in PBS solution and PBS were added into 3 wells in a 6-well plate. Isolated hiPSC-CM and NHCF were suspended in 500 μL of PBS after centrifugation and added to a 6-well culture insert with a 3 μm pore membrane. The insert was immersed in the FN solution and agitated at 500 rpm for 1 min at room temperature using a MixMate shaker (Eppendorf). After FN coating, the cells were washed with PBS. Next, the cells were immersed in the G solution and agitated again under the same conditions as FN. After the G coat, the cells were washed with PBS. These steps were considered to be one cycle and four cycles were completed. Finally, the cells were coated with an FN coat. (FN: five times, G: four times).

### Fabrication of orientation-controlled 3D cardiac tissues using a cell accumulation technique

After the LbL coating process, FN-G nanofilms were formed on each cell membrane. The 5 mL cell suspension mixed with LbL coated hiPSC-CM (5.4 × 10^6^ cells) and NHCF (1.8 × 10^6^ cells) were seeded into the 6-well culture inserts which were set in a 6-well plate. An HBC gel frame coated with fibronectin and 2 mL culture medium was added to the outside of the culture insert. After 2 h culture, 5 mL medium was added into the well to connect the inner and outer of the insert medium in the culture insert. It was then incubated in 5% CO_2_ at 37 °C. In the case of fabrication of 3D cardiac tissue with blood capillaries, 5 mL cell suspension mixed with ECM nanofilm-coated hiPSC-CM (5.4 × 10^6^ cells) and NHCF (1.8 × 10^6^ cells) and uncoated HMVEC (7.1 × 10^5^ cells) were seeded into the 6-well culture inserts which were set in a 6-well plate. An HBC gel frame coated with fibronectin and 2 mL culture medium was added to the outside of the culture insert. After 2 h culture, 5 mL medium was added into the well to connect the inner and outer of the insert medium in the culture insert. It was then incubated in 5% CO_2_ at 37 °C. To compare cell morphology with shape controlled 3D cardiac tissue, uncontrolled 3D cardiac tissue was fabricated by using 24-well culture insert. The 400 μL cell suspension mixed with LbL coated hiPSC-CM (0.75 × 10^6^ cells) and NHCF (0.25 × 10^6^ cells) were seeded into the 24-well culture inserts which were set in a 24-well plate. In the case of 3D cardiac tissue with vascular network, the 400 μL cell suspension mixed with LbL coated hiPSC-CM (0.75 × 10^6^ cells), NHCF (0.25 × 10^6^ cells) and HMVEC (1.0 × 10^5^ cells) were seeded into the 24-well culture inserts which were set in a 24-well plate. Add 1 mL culture medium to the outside of the culture insert. After 2 h culture, 1 mL medium was added into the well to connect the inner and outer of the insert medium in the culture insert. It was then incubated in 5% CO_2_ at 37 °C.

### Fluorescence staining of 3D cardiac tissues and microscopy

For the evaluation of the 3D cardiac tissues, the samples were washed with PBS, fixed with 4% PFA and permeabilized with 0.2% Triton X for 20 min at room temperature. After washing with PBS, samples were blocked with 1% bovine serum albumin (BSA) for 1 hour. The blocked samples were incubated with primary antibodies at 4 °C overnight. The samples were subsequently incubated with secondary antibodies at room temperature for 2 hours. F-actin was stained with Acti-stain 488 fluorescent for 60 min at room temperature in darkness. cTnT was stained with monoclonal anti-cTnT antibody (1:300) as the primary antibody and Alexa 546 conjugated anti-mouse IgG antibody (1:200) or Alexa 488 conjugated anti-mouse IgG antibody (1:200) as the secondary antibody. CD31 staining, was used polyclonal anti-CD31 antibody (1:200) as the primary antibody and Alexa 546 conjugated anti-rabbit IgG antibody (1:200) as the secondary antibody. Following this, samples washed with PBS and stained the cell nuclei with DAPI (10 μg/mL) for 30 min at room temperature in darkness. The stained samples were observed by confocal laser scanning microscopy (CLSM) (FLUOVIEW FV10i: Olympus) (LSM 780: Carl Zeiss).

### Evaluation of cardiac tissue cell orientation and contractile properties

ImageJ plug-in, OrientationJ, was used for the quantification of the fabricated 3D tissue’s cell orientation^63,64^. This plug-in showed the directional distribution of the actin fibers and vascular networks from observed fluorescence staining images. Moreover, we tried to assess the contractile properties of the fabricated 3D cardiac tissue by image analysis with the ImageJ plug-in, particle image velocimetry (PIV)^62,65^. This method calculates the cross correlations between two consecutive images divided into small blocks and shows the velocity and direction of contraction.

### Statistical analysis

The contractile properties of orientation-controlled 3D cardiac tissue (n = 3) and uncontrolled 3D cardiac tissue (n = 3) were evaluated from the PIV analysis. These data were expressed as means ± standard deviation (SD). Comparisons between each tissue contraction velocity and relaxation velocity were performed by a Student’s t-test. A *p* value less than 0.05 was considered be statistically significant.

## Supplementary information

Supplementary Information.

Supplementary Movie 1

Supplementary Movie 2

Supplementary Movie 3
