# Supplementary material for: Vascularized cardiac tissue construction with orientation by layer-by-layer method and 3D printer
Source: Sci Rep. 2020 Mar 26;10:5484. doi: 10.1038/s41598-020-59371-y (PMC7098983; doi:10.1038/s41598-020-59371-y)

Vascularized cardiac tissue construction with orientation by layer-by-layer method and 3D printer

Authors:

Yoshinari Tsukamoto, Takami Akagi, and Mitsuru Akashi*

Affiliations and contact information:

Building Block Science Joint Research Chair, Graduate School of Frontier Biosciences, Osaka University, 1-3 Yamadaoka, Suita 565-0871, Japan

*Corresponding author:

Mitsuru Akashi

Building Block Science Joint Research Chair, Graduate School of Frontier Biosciences, Osaka University, 1-3 Yamadaoka, Suita 565-0871, Japan.

Tel: +81-6-6105-5247, Fax: +81-6-6878-9712

E-mail address: akashi@fbs.osaka-u.ac.jp

Supplementary figure legends

Supplementary Figure 1. The overview image of shape controlled 3D cardiac tissue. The fluorescent image indicated that the cells in the 3D tissue were oriented in the two points. In the fluorescence image, green indicates F-actin and red indicates cardiac troponin T (cTnT). The bright field image shows three points in the 3D cardiac tissue. In the bright field image, the blue frame indicates HBC gel and the red frame indicates 3D cardiac tissue.

Supplementary Figure 2. The fluorescent image of 3D cardiac tissue image stained with fluorescent labeling phalloidin (F-actin) (green) and anti-cardiac troponin T (cTnT) antibody (red). The 3D cardiac tissues of shape controlled and uncontrolled were obtained by confocal microscope using a 10× and 60× objective lens.

Supplementary Figure 3. The picture of dispenser type 3D printer. (a) The picture shows that X-Z moving motors module and Y moving stage. (b) The picture shows that Peltier element and Dispensing nozzle.

Supplementary Movie 1. The original movie of beating behavior of orientation-controlled 3D cardiac tissue.

Supplementary Movie 2. The original movie of beating behavior of uncontrolled 3D cardiac tissue.

Supplementary Movie 3. The movie of HBC gel frame fabricated using 3D printer.

Supplementary Figure 1.

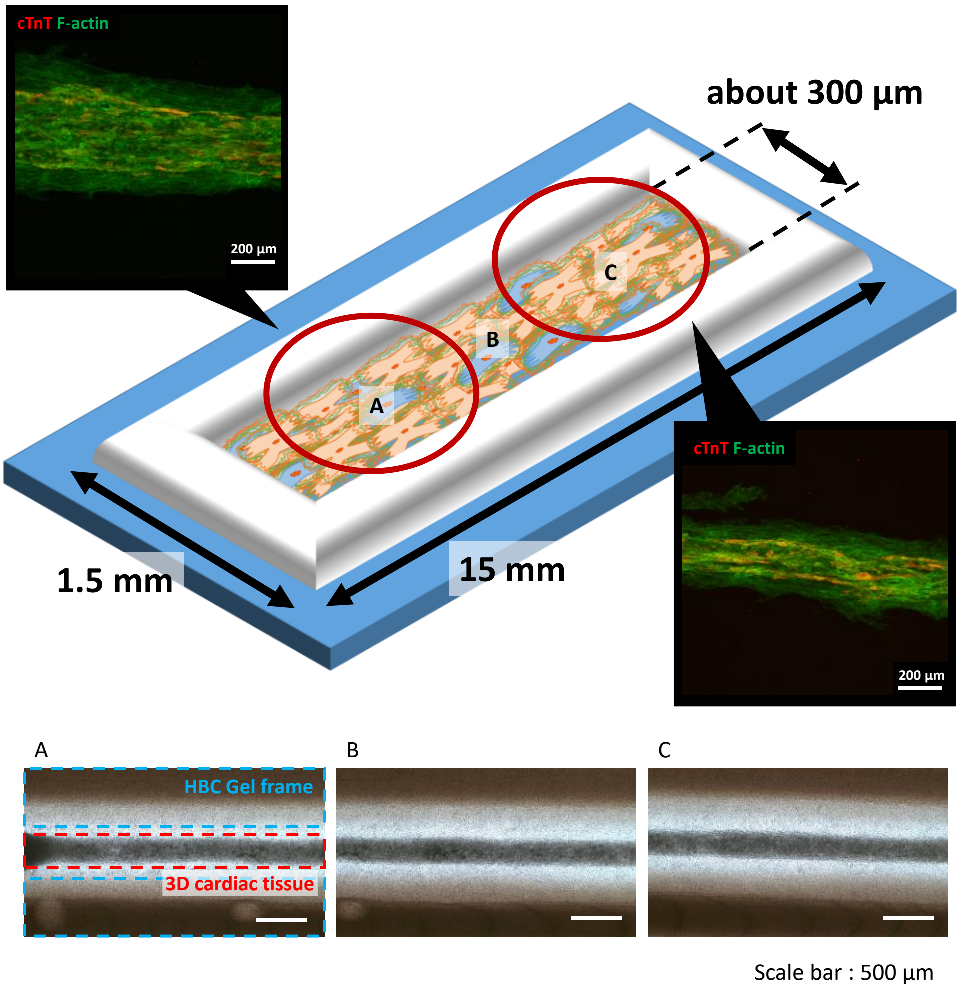

Supplementary Figure 2.

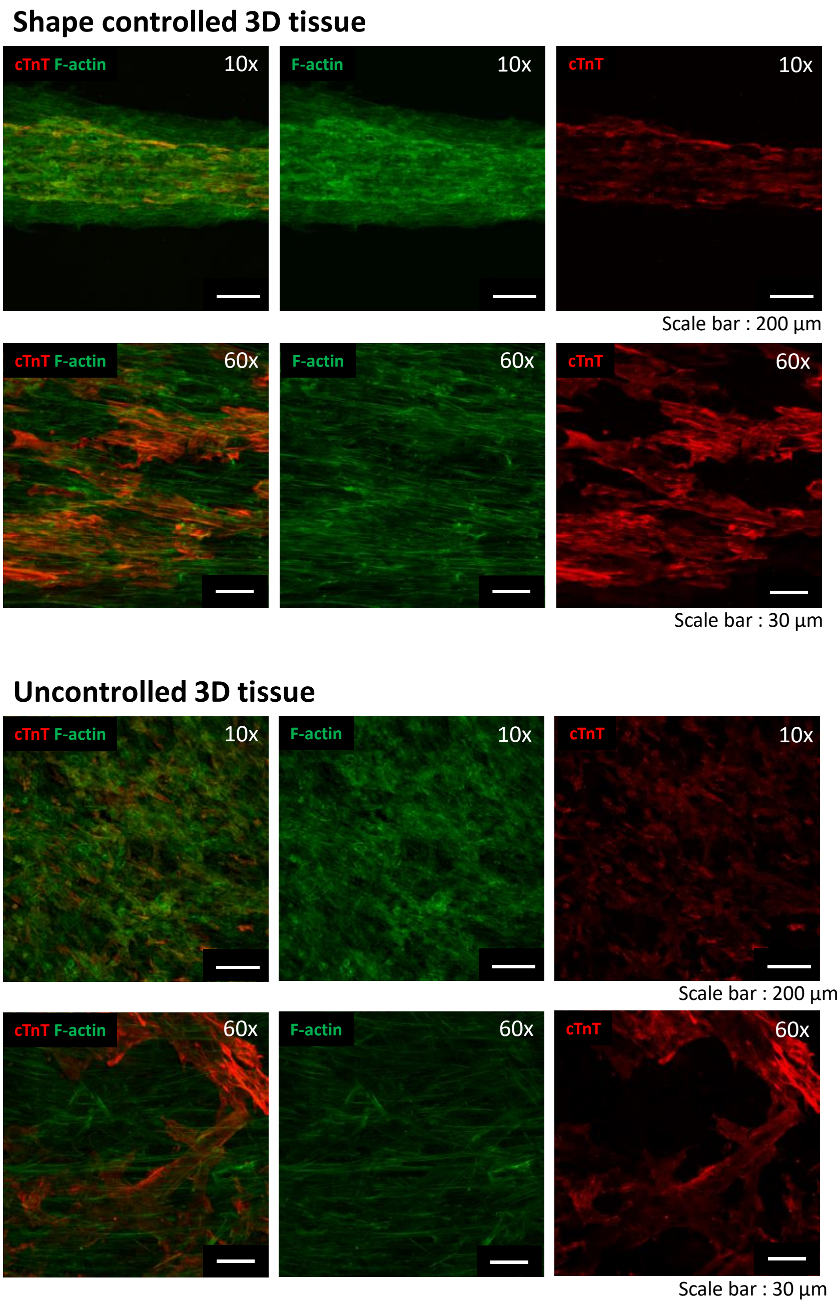

Supplementary Figure 3.

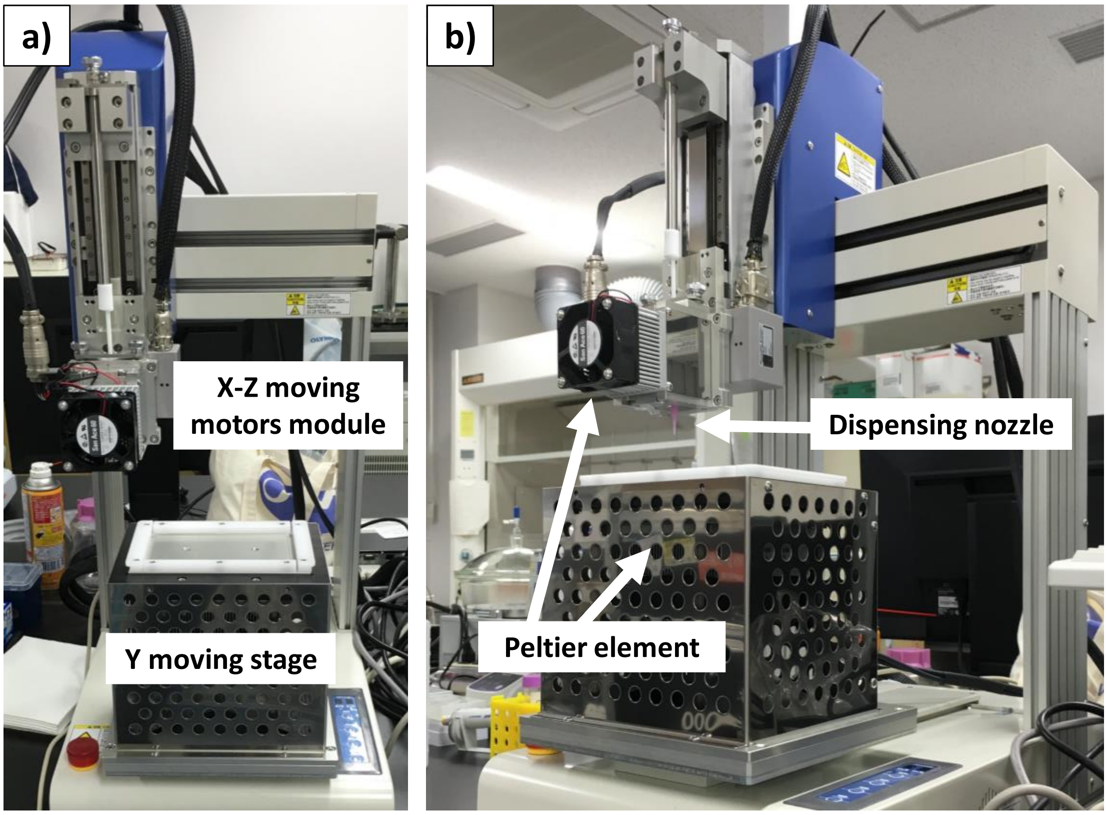

Supplement: Supplementary file 1 — Supplementary Information. [file 41598_2020_59371_MOESM1_ESM.pdf]
